# Management of rare and undiagnosed diseases: insights from researchers and healthcare professionals in Türkiye

**DOI:** 10.3389/fpubh.2024.1501942

**Published:** 2025-01-15

**Authors:** Sinem Durmus, Emrah Yucesan, Sinem Aktug, Begum Utz, Ahmet Okay Caglayan, Pinar Gencpinar, Cagatay Günay, Yavuz Oktay, Ravza Nur Yildirim, Ayca Yigit, Ugur Ozbek

**Affiliations:** ^1^Department of Medical Biochemistry, Faculty of Medicine, İzmir Katip Çelebi University, Izmir, Türkiye; ^2^Institute of Neurological Sciences Department of Neurogenetics, Istanbul University-Cerrahpasa, Istanbul, Türkiye; ^3^Izmir Biomedicine and Genome Center, Izmir, Türkiye; ^4^Department of Medical Genetics, Faculty of Medicine, Dokuz Eylül University, Izmir, Türkiye; ^5^Department of Pediatric Neurology, Faculty of Medicine, Izmir Katip Celebi University, Izmir, Türkiye; ^6^Department of Pediatric Neurology, Faculty of Medicine, Dokuz Eylül University, Izmir, Türkiye

**Keywords:** RareBoost, rare disease, survey, Türkiye, undiagnosed disease, unmet needs

## Abstract

**Introduction:**

Diagnosis, treatment and management of rare diseases (RD) pose unique challenges due to their complex nature, significantly impacting the daily experiences of researchers and healthcare professionals working in this field. Despite increasing awareness and progress in the field of RD worldwide in recent years, a significant gap remains in our understanding of the specific barriers that these professionals face in their work with RD. This study provides a detailed survey analysis that sheds light on the challenges that researchers and healthcare professionals face in diagnosing, treating, managing and conducting research on RD.

**Methods:**

We developed a national online survey with three RD stakeholder groups (Researchers, Healthcare professionals and researcher-healthcare professionals) to identify the main challenges and needs in Türkiye for the diagnosis, treatment and follow-up processes of rare and undiagnosed diseases.

**Results:**

The survey was completed by 363 participants, revealing that participants face key challenges such as the need to refer patients to specialized centers, financial burdens, limited access to necessary tests, inadequate support for rare disease research and a lack of interdisciplinary collaboration. Participants also noted that RD are inherently difficult to conduct research on with small cohorts. Survey results also suggest a number of policy improvements to accelerate research on RD: increased funding, establishment of robust surveillance systems, and development of comprehensive national action plans and guidelines on RD.

**Discussion:**

To the best of our knowledge, this is the first study to be conducted in Türkiye. This study contributes to the understanding of the needs of professionals in rare disease research and highlights the urgent need for system improvements to support them.

## 1 Introduction

Depending on the criteria set by different countries, some diseases are considered “rare” and these “rare” conditions are collectively referred to as Rare Diseases (RD) (1). Currently, it is thought that RD affect almost 25–30 million individuals in the United States of America (USA) and about 30 million in the European Union (EU) (2, 3). A review of the data from the OMIM (Online Mendelian Inheritance in Man), a highly reliable human diseases database, indicates that there are approximately 7,000 distinct RD. In a 2019 study, the ORPHANET database was studied with a reported 6,172 RD (4, 5). As indicated in the aforementioned study, 71.9% of these diseases have a genetic basis, and 69.9% manifest during childhood (4). Approximately 35% of deaths among live births in the first year of life are attributable to conditions caused by neurodevelopmental disorders (6). Of note is that approximately one-third of children born with RD die before reaching the age of five (7). A total of 149 RD have been identified, representing 4.2% of all disease groups, with a prevalence of 1–5/10,000 in the population. Of the RD, 84.5% encompass 5,304 diseases, with a prevalence of less than 1/1,000,000 (4).

RD constitute a diverse group of diseases that manifest in different forms and occur at varying rates in different geographical regions worldwide (4). Their low prevalence leads to a lack of information about the disease(s) and a low number of experts, as well as limited and challenging access to these experts (8). Considering the aforementioned information, it is evident that combating these illnesses represents a significant challenge for patients and their families, as well as a considerable burden on the social and health policies of states (9).

To map unmet needs and identify potential avenues for further research in the field of rare and undiagnosed diseases, we present survey data gathered from researchers and healthcare professionals under a work package within the EU’s Horizon 2020 Research and Innovation-supported ERA Chair project “*RareBoost*.” The RareBoost project is an ERA Chair-funded initiative implemented by Izmir Biomedicine and Genome Center (IBG). The primary objective of the project is to establish IBG as an internationally recognized center of excellence in innovation and research and development. Furthermore, within the scope of RareBoost, the professional development of researchers is emphasized through improving their soft and career skills in management, innovation and administration, optimizing their education and training qualifications, and developing their research and innovation capacity in the field of RD. Additionally, the RareBoost project will enable greater knowledge transfer at the national and international levels and raise awareness of RD through effective science communication. The main goals of the survey were to evaluate the current level of awareness about RD within the scope of Türkiye, document the challenges faced by various groups of stakeholders, and propose some potential solutions for future improvements. In recent years, many activities have been carried out in Turkey to raise awareness on Rare Diseases. To give an example of some of these; in Türkiye, especially in the last 10 years, a large number of events have been held in foundation or state universities and various hospitals on the last day of February every year to raise awareness of rare diseases. Again, as a positive result of these awareness raising activities in Türkiye, the ‘Department of Autism, Mental Special Needs and Rare Diseases, which is affiliated to the Ministry of Health and was established specifically for the coordination of rare disease research, started its activities in 2020.^1^ Following the rare diseases event organized by the Presidency of Health Institutes of Türkiye under the Ministry of Health, a final statement titled ‘Rare Diseases Awareness Day Symposium Report’ was published by the Public Health and Chronic Diseases Institute of Türkiye in March 2021. In parallel with these developments, in 2024, 7 rare disease-related family associations came together to form the ‘Rare Diseases Federation’.^2^ As mentioned above, Rare Disease studies in Türkiye continue at local level. This study represents, to the best of our knowledge, the first and one of the most comprehensive surveys focused on researchers and healthcare professionals working on RD in Türkiye. RD is crucial public health and social issue in Türkiye, because of high consanguinity. It is expected that the findings will offer a critical basis for overcoming challenges and developing solutions in this area.

## 2 Materials and methods

### 2.1 Survey design and participant recruitment

The survey was developed based on literature and different surveys from other global regions using the search terms “rare diseases + undiagnosed diseases + survey” on Pubmed (10–12). After question selection, a team of medical doctors from different specialties (neurologists, pediatricians, pharmacologists, clinical geneticists, etc.), molecular geneticists, bioinformatics, patient organization representatives, and medical school and/or life sciences students were asked to evaluate the questions. A survey development software, SurveyMonkey (13), was used to construct the survey.

The RareBoost team developed a national survey with three RD stakeholder groups to identify the main challenges and needs in Türkiye for the diagnosis, treatment and follow-up processes of rare and undiagnosed diseases, and to provide a roadmap for future research projects based on the needs identified by the below groups:

1. Researchers
2. Healthcare professionals
3. Both researchers and healthcare professionals (Researcher-healthcare professionals)

The first group (researchers) consisted of researchers such as molecular biologists and bioinformaticians. The second group (healthcare professionals) consisted of healthcare professionals such as pediatricians, neurologists, clinical geneticists, pharmacologists, who stated that they were not involved in research but only in patient care. The last group (Researcher-healthcare professionals) was consisted of the intersection set of the first and second groups. Participants in the second and third groups answered all questions, whereas participants in the first group were exempted from questions asking about direct interactions with the patient (Q12, Q13, Q14, Q15, Q17, Q18, Q19, Q20, Q21).

The survey was designed to be completed in 7 to 10 min and included demographic questions about participants, such as age, specialty, and duration of employment in their respective fields of expertise. The survey consisted of 35 multiple choice questions as well as a consent statement to participate in the online survey. The survey to allowed participants an additional text box to provide free text responses to questions where they selected “other.” Some questions asked healthcare professionals and researcher-healthcare professionals about challenges specific to these areas, which are described in the text. Experts in this field and a linguist evaluated the survey to ensure that the questions were clear and understandable before it was sent to participants. The survey was distributed by the research team via email, social media (Instagram, LinkedIn, Facebook, and WhatsApp), and the RareBoost project website.^3^ The survey was conducted in Turkish and was open for 9 weeks, completed at the end of January 2024. Response to all 36 questions were not mandatory.

### 2.2 Ethical governance

This study was conducted in accordance with the Declaration of Helsinki guidelines and approved by the Izmir Biomedicine and Genome Center (IBG) Ethics Committee (protocol number: 2023–039). After reading a summary of the General Data Protection Regulation (GDPR) and the conditions for participation in this study (Supplementary material 1), participants were asked to indicate their consent. Only those who granted their consent after reading the GDPR document were given access to the survey questions.

### 2.3 Data collection and analysis

Survey responses were recorded via the SurveyMonkey platform, which was also used to create the questionnaire. Data was then imported into SPSS V22 for statistical analysis. Prior to statistical analysis by the RareBoost research team, typographical and punctuation errors were rectified. Descriptive analysis is presented in the form of numbers, percentages and 95% confidence intervals (CI). A Z-test for proportions and post-hoc pairwise comparisons were used to explore specific group differences. Participants’ responses to the question regarding the general awareness of RD were quantified using a weighted scoring system, with each awareness level assigned a numerical score: “Very Low” = 1, “Low” = 2, “Medium” = 3, “High” = 4, and “Very High” = 5. The mean awareness score was calculated, which served as a single measure representing the general level of awareness in the study. The calculation was performed by multiplying the frequency of responses for each category by the corresponding score, summing these values and dividing by the total number of responses.

## 3 Results

A total of 363 people participated in the survey, which consisted of 36 questions divided into six sections: demographic characteristics and professional background (section 1), experience (section 2), challenges in diagnostic processes (section 3), patient care and support of patients and their relatives (section 4), research and education (section 5) and stakeholders in the ecosystem and collaborative efforts (section 6).

### 3.1 Participant demographics and professional background

Of note is the disproportionate representation of women (65.84% vs. 33.88%) in the survey. Respondents were generally experienced and mid-career in the RD field, with over half having more than 15 years of experience (Table 1). The majority were specialists in Pediatrics (39%) and Biology-Genetics (26%; Supplementary Table 1). More than 80% of respondents indicated that their institution was involved in RD activities, with the most prevalent activity being diagnosis and genetic testing (82.04%). Participation in treatment and management of patients with RD and in research and clinical trials was also high (Supplementary Figure 1A). The least selected response was collaboration with patient associations/NGOs (27.82%). Those who did not have RD activities at their institution often attributed this to the necessity of referral due to insufficient testing capacity (Supplementary Figure 1B). Ten participants who selected the “Other” option cited reasons such as a lack of data, inadequate laboratory infrastructure, lack of patient referrals, lack of a specialized unit in their hospitals, difficulties in maintaining patient records due to high patient traffic, the high cost of tests in private hospitals and/or a lack of specialists for RD. The survey participants were stakeholders from various regions of Türkiye, ensuring broad geographical representation. However, specific details regarding the exact locations or institutions where respondents work were not collected as part of the survey.

**Table 1:** Participant demographics and professional background.

Response	n	Distribution % (95% CI)
Q2-Sex (*n* = 363)		
Male	123	33.88 (29.21–38.90)
Female	239	65.84 (60.82–70.53)
Decline to comment	1	0.28 (0.05–1.54)
Q3-Age range (*n* = 363)		
18–24	7	1.93 (0.94–3.93)
25–29	35	9.64 (7.01–13.11)
30–39	108	29.75 (25.28–34.65)
40–49	111	30.58 (26.06–35.50)
50–59	64	17.63 (14.06–21.88)
60 or above	38	10.47 (7.72–14.04)
Q4-Professional information (*n* = 363)		
Researcher	113	31.13 (26.37–35.89)
Healthcare Professional	113	31.13 (26.37–35.89)
Researcher–Healthcare Professional	137	37.74 (32.75–42.73)
Q6-Subspecialty (*n* = 363)		
Yes	174	47.93 (42.84–53.07)
No	189	52.07 (46.93–57.16)
Q7-Duration of work in area of expertise* (*n* = 363)		
< 1 year	16	4.41 (2.73–7.04)
1–5 years	78	21.49 (17.57–26.00)
6–10 years	61	16.80 (13.31–20.99)
11–15 years	60	16.53 (13.06–20.70)
>15 years	148	40.77 (35.84–45.90)
Q8-Affiliated institution/organization (*n* = 363)		
University hospital (public or private)	182	50.14 (45.02–55.25)
Training-research hospital	49	13.50 (10.36–17.40)
Public hospital	12	3.31 (1.90–5.69)
Private hospital	9	2.48 (1.31–4.64)
University / research institute	107	29.48 (25.02–34.36)
Non-governmental organization (association, foundation, etc.)	2	0.55 (0.15–1.99)
Other	2	0.55 (0.15–1.99)
Q9-Status of activities in the field of RD in participants institutions (*n* = 363)		
Yes	293	80.72 (76.66–84.77)
No	70	19.28 (15.23–23.34)

Q, Question number in survey; *Including assistantship/specialization or PhD period; Response rate for Q1–Q9: 100%.

### 3.2 Participants’ working experiences

This section was designed only for healthcare professionals and researcher-healthcare professionals (250 participants), as detailed Table 2. Approximately 50% of the participants stated that they encounter 11–50 RD cases annually, while over 80% see at least three RD cases annually. However, the proportion of respondents encountering 11–50 cases per year decreased significantly during the follow-up period. Responses to Q14 reported that 70% of participants are able to conduct regular follow-ups with their patients (*p* < 0.001). The primary reason for the inability to follow up regularly was the referral of patients to advanced centers (44.44%) and patients’ failure to proceed with the follow-up (38.89%). In the “Other” category, respondents indicated that they were unable to follow up with patients for a number of reasons, including the completion of rotations, a lack of referral permission, an inability to follow up due to specialization and the provision of laboratory services only.

**Table 2:** Experiences.

	n	Distribution % (95% CI)
Q12-Encounter frequencies with RD cases (*n* = 237)		
Frequently (11–50 cases per year)	118	49.79 (43.48–56.11)
Sometimes (3–10 cases per year)	72	30.38 (24.88–36.51)
Rarely (1–2 cases per year)	39	16.46 (12.28–21.70)
Never	8	3.38 (1.72–6.52)
Q13-Encounter frequencies with RD cases during the follow-up process (*n* = 237)		
Frequently (11–50 times per year)	81	34.18 (28.43–40.43)
Sometimes (3–10 times per year)	89	37.55 (31.63–43.87)
Rarely (1–2 times per year)	52	21.94 (17.14–27.63)
Not follow up	15	6.33 (3.87–10.18)
Q14-Capability for regular patient follow-up (*n* = 237)		
Yes	164	69.20 (63.05–74.73)
No	73	30.80 (25.27–36.95)
Q15-The Reasons for the inability to regularly follow-up patients (*n* = 72)		
Referring to advanced centers	32	44.44 (33.54–55.91)
Patients’ failure to proceed the follow-up	28	38.89 (28.47–50.44)
Patient is referred to and followed up by appropriate departments	9	12.50 (6.72–22.08)
Other	10	13.89 (7.72–23.71)

Response rate for Q12–Q14: 94.80% and for Q15: 98.63%.

### 3.3 Challenges in diagnostic processes

**Figure 1:**
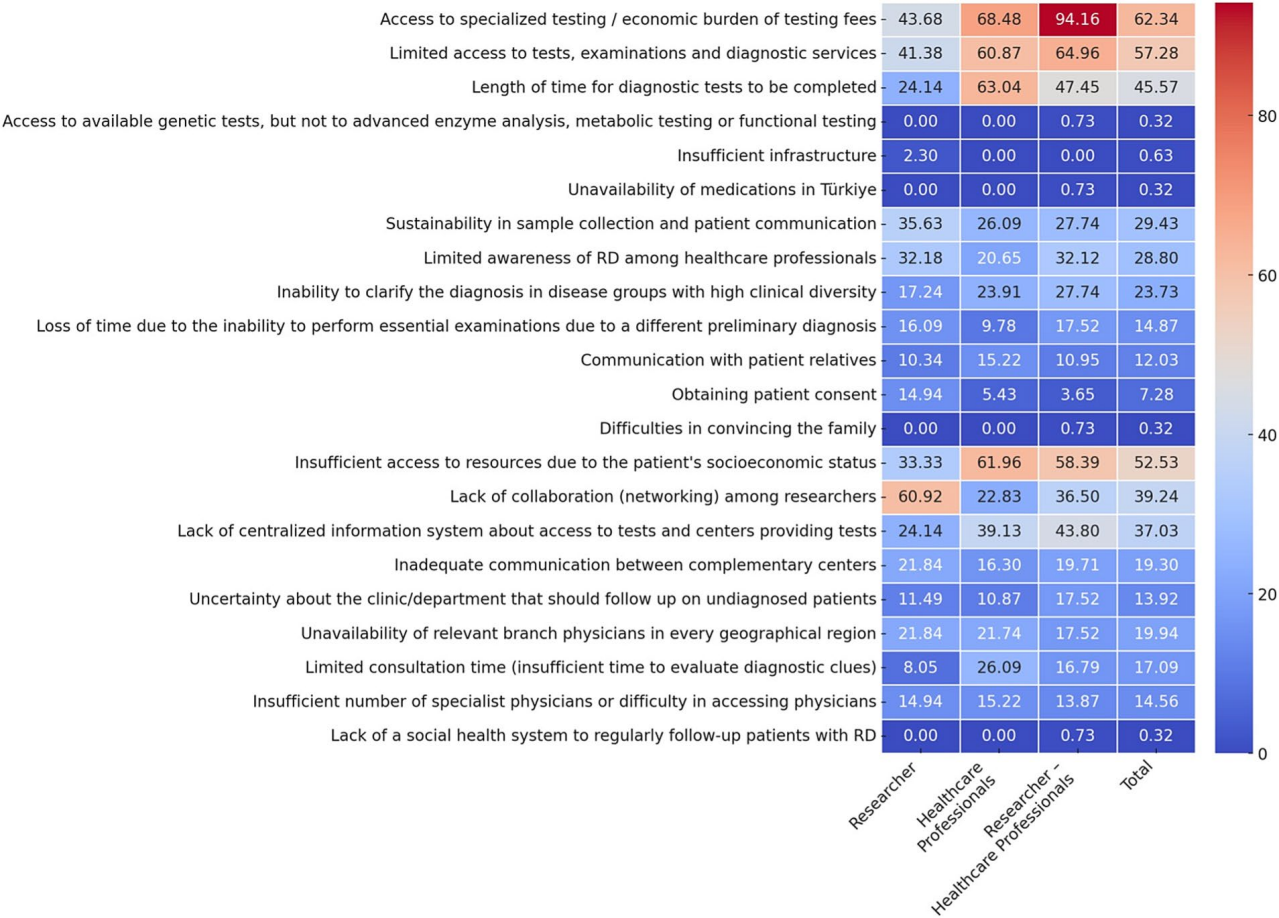
Distinct challenges faced by different professional groups in the management of RD (Q16). Data are presented as percentages. Multiple options could be selected in this question (up to a maximum of 7). Response rate: 87.05% for total, 60.0% for healthcare professional, 76.99% for researcher, 93.88% for physician, 93.43% for researcher-physician. *Difficulties in convincing the family as a result of wrong/incomplete genetic counseling and evaluations by different departments, resulting in the family receiving inaccurate/incomplete diagnosis or treatment ‘Access to special tests / economic burden of test fees’, it is intended to emphasize the inaccessibility of special tests due to financial inadequacy.

The five most frequently reported challenges in the management of rare and undiagnosed diseases, based on all respondents, was access (or lack of) to specialized tests and the economic burden of test fees (62.34%), limited resources, lack of access to examinations and diagnostic tests (57.28%), insufficient access to resources due to the patient’s socioeconomic status (52.53%), length of time for diagnostic tests to be completed (45.57%) and a lack of collaboration (networking) among researchers (39.24%). However, when stakeholder groups are analyzed separately, it becomes clear that different groups are more likely to face different challenges (Figure 1).

Top Challenges for Researchers: The two most prevalent challenges identified were a lack of collaboration (networking) among researchers (60.92%) and the economic burden associated with limited access to specialized testing (43.68%). Furthermore, 41.38% of researchers identified limited access to tests, examinations and diagnostic services as a significant challenge (Figure 1).

Top Challenges for Healthcare Professionals: The economic burden and access to specialized testing were identified as the most common problem in accessing specialist tests, with 68.48% of respondents indicating this as a significant issue. Subsequently, respondents identified length of time diagnostic test to be completed (63.04%) and inadequate access to resources due to the patient’s socioeconomic status (61.96%) as significant challenges (Figure 1).

Top Challenges for Researcher-Healthcare Professionals: The overwhelming majority of this group (94.16%) identified access to specialized tests and the economic burden of testing fees as the most significant challenge. Subsequently, 64.96% of respondents identified limited access to diagnostic tests, while 58.39% highlighted the impact of the patient’s socioeconomic status on their ability to obtain tests (Figure 1).

Among 250 healthcare professionals and researcher-healthcare professionals, 229 responded to the question whether there was an interdisciplinary council for RD assessment in their center (Table 3). The rates were similar between those who reported that the council existed (43.23%) and those who did not (45.85%), both of which were higher than the rate among those who did not know (*p* < 0.001, for both). A significantly higher proportion of participants (77.29%) reported having access to RD diagnostic resources (such as radiologic, metabolic and genetic testing) as opposed to those who did not (22.71%; *p* < 0.001). The most frequently reported method of access was referral services, accounting for 83.05% of cases. The next most common sources of funding were the Ministry of Health (41.24%), drug companies (30.51%), and project-supported (31.07%) sources, which were all significantly less common than the referral option (*p* < 0.001, for all). While Ministry-supported tests are utilized more frequently than those supported by drug companies and projects, the accessibility levels of the latter two are comparable. Our findings indicate that the most significant obstacle to accessing diagnostic resources for RD is the insufficient availability of tests in institutions (86.27%). This is significantly more prevalent than issues pertaining to general health insurance coverage (*p* = 0.011) and the absence of family insurance (*p* < 0.001).

**Table 3:** Challenges encountered during the diagnosis process.

	n	Distribution % (95% CI)
Q17-The need to refer patients to other centers (public/private) for clinical evaluation and/or tests required for the diagnosis of RD (*n* = 229)		
Yes	174	75.98 (70.05–81.06)
No	55	24.02 (18.94–29.95)
Q18-Availability of interdisciplinary councils for RD evaluation (*n* = 229)		
Yes	99	43.23 (36.78–49.69)
No	105	45.85 (39.34–52.36)
Do not know	25	10.92 (6.91–14.93)
Q19-Access to resources for diagnosing RD (such as radiological, metabolic, and genetic tests) (*n* = 229)		
Yes	177	77.29 (71.59–83.00)
No	52	22.71 (17.00–28.41)
Q20-The resources I have access to for diagnosing RD are… (*n* = 177)		
Social Security Insurance-supported diagnostic tests	73	41.24 (34.25–48.61)
Drug company-supported resources	54	30.51 (24.20–37.65)
Project-supported access	55	31.07 (24.72–38.23)
Referral to appropriate centers for unavailable tests	147	83.05 (76.84–87.86)
Q21-The reasons for the lack of access to resources for the diagnosis of RD are... (*n* = 51)		
The tests covered by general health insurance are insufficient	33	64.71 (50.99–76.37)
The family does not have any insurance	9	17.65 (9.57–30.25)
The tests available at the institution are insufficient	44	86.27 (74.28–93.19)

For Q20, participants were allowed to select more than one option. Response rate for Q17–Q19: 91.60, 100% for Q20, 98.08% for Q21.

### 3.4 Patient care and support of patients and their relatives

**Figure 2:**
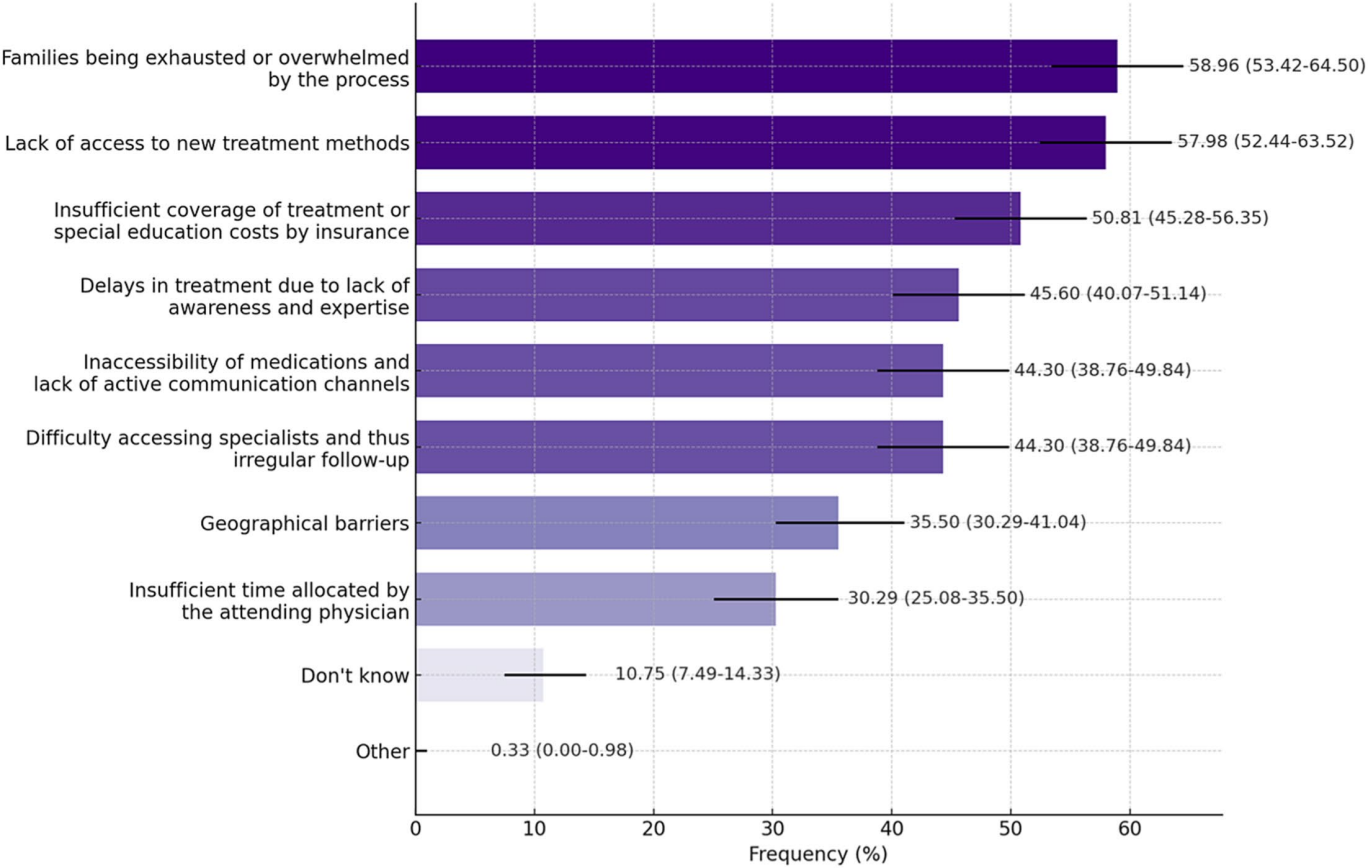
Challenges encountered in patients’ access to specialized care and treatments (Q24). Data are presented as percentages with 95%CI. Participants were allowed to select more than one option. Response rate: 84.57%.

**Figure 3:**
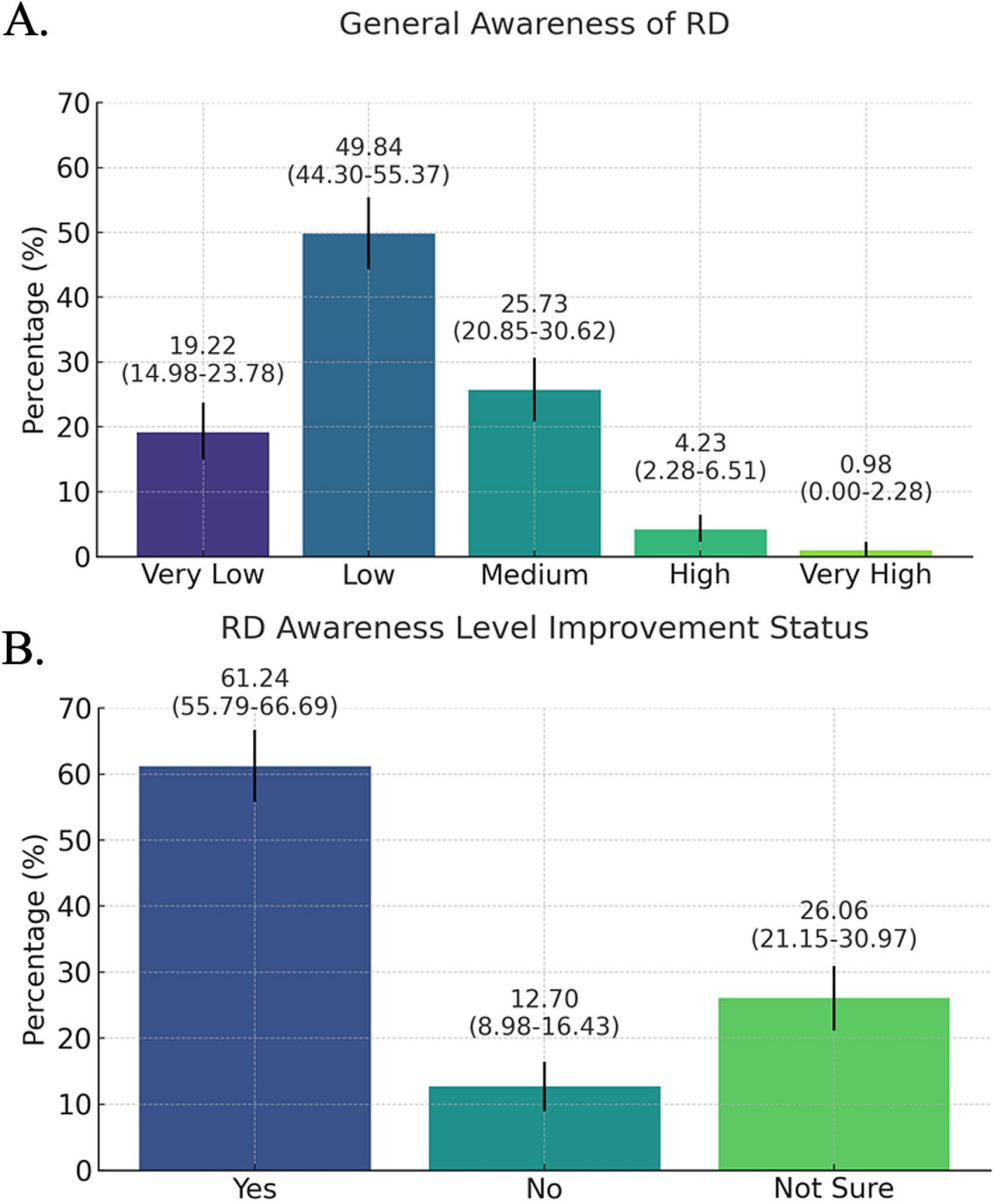
Participants’ responses to **(A)** “How would you rate the general awareness of rare diseases?” (Q25) and **(B)** “Do you think the level of awareness of rare diseases has improved in recent years?” (Q26). Data are presented as percentages with 95%CI. Response rate: 84.57% for both.

There was no significant difference in responses regarding whether support was provided to meet patients’ needs during the diagnosis and follow-up process; participants who reported receiving support most frequently mentioned follow-up/genetic counseling, rehabilitation, communication with representatives, and services from specialized clinics (Supplementary Table 2). According to participants, the most common challenges patients encountered in accessing specialized care and treatments are families being exhausted or overwhelmed by the process (58.96%), lack of access to new treatment methods (57.98%), and insufficient insurance coverage (50.81%). For participants who chose the ‘other’ option, a box was opened for them to provide an explanation, and they wrote: *‘We have to admit patients from remote areas for tests and we do not have enough beds. We often lack special units for socio-economic and psychological support.”* It is also noteworthy that approximately 11% of the participants chose the “I do not know” answer for this question (Figure 2). Figure 3A shows that participants exhibited a general lack of awareness of RD. The mean awareness score was 2.18, indicating low awareness of RD. This indicates a necessity for enhanced educational programs to improve comprehension of RD. The majority of participants (61.24%) indicated that awareness of RD has increased in recent years (Figure 3B).

### 3.5 Research and education

**Figure 4:**
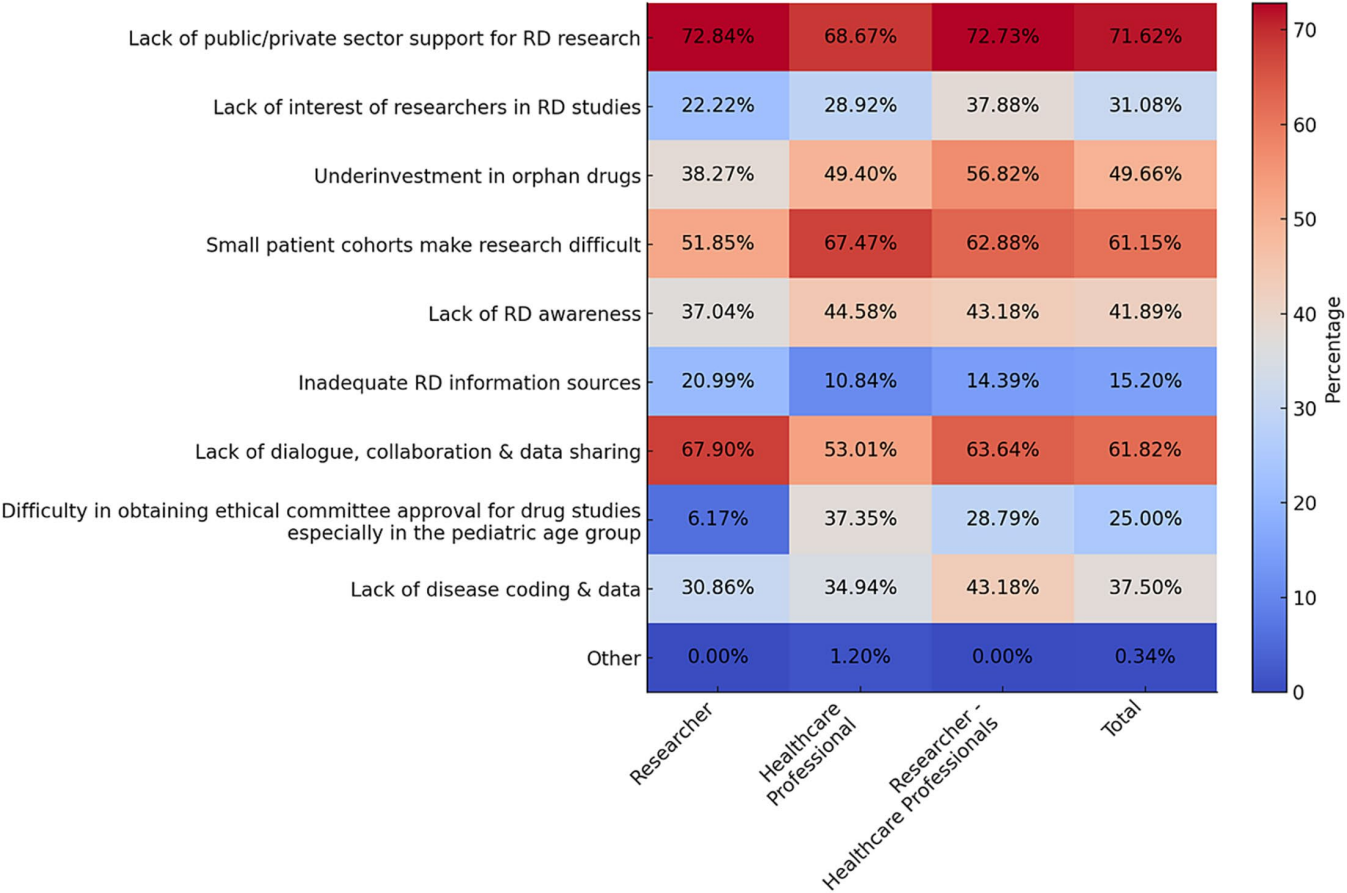
Challenges faced by all stakeholder groups and total participants in scientific research on RD and orphan drugs in Türkiye (Q32). Data are presented as percentages. Multiple options could be selected in this question (up to a maximum of 7). Response rate: 81.54% for total, 53.33% for healthcare professional, 71.68% for researcher, 84.69% for physician, 90.51% for researcher-physician.

Most participants reported being involved in studies related to and receiving training in RD (*p* < 0.001 for both questions). The scope of these studies is shown in Supplementary Table 3. To stay updated on RD, the majority of respondents frequently use professional scientific events (83.71%) and scientific publications (81.43%; Supplementary Figure 2). The majority of participants were willing to share research data. Approximately half (52.35%) agreed to do so for interdisciplinary studies under specified protocols (Supplementary Figure 3). Despite the numerous challenges faced in scientific research on RD and orphan drugs, all three stakeholder groups, with remarkable consensus, highlighted the same three challenges albeit in different orders (Figure 4). The most striking finding was the widespread concern over the lack of public/private sector support for RD research. The second most significant challenge for healthcare professionals is the difficulty in conducting research due to small patient cohorts, while researchers and researcher-healthcare professionals ranked this third, placing lack of dialog, collaboration and data sharing as the second major issue.

### 3.6 Stakeholders in the ecosystem and collaborative efforts

Differences were observed among participants regarding the frequency of collaboration with other healthcare professionals diagnosing RD; those who answered “Rarely” were significantly more than those who answered “Daily” or “Monthly” (*p* = 0.005 and *p* = 0.020, respectively). Furthermore, 15.15% of respondents indicated that they had never engaged in any collaborative endeavors (Supplementary Table 4). Approximately 61.95% of respondents indicated that they had not been involved with or supported institutions or NGOs related to RD. Additionally, 70.03% of respondents reported being unaware of government policies on the subject (*p* < 0.001). Moreover, 74.14% of participants indicated that national and international funding support should be enhanced, while 68.62% underscored the necessity for follow-up systems for RD patients and 63.45% advocated for the formulation of national action plans and guidelines (Table 4).

**Table 4:** Q36-Suggested policy improvements in the field of rare and undiagnosed diseases (*n* = 290).

Responses	n	Distribution % (95% CI)
Increasing national/international funding support for pharmaceutical, advanced research, and research projects	215	74.14 (69.31–78.97)
Establishing follow-up systems for RD patients	199	68.62 (63.10–73.79)
Developing national action plan & guidelines on RD	184	63.45 (57.93–68.97)
Expansion of prenatal screening coverage	158	54.48 (48.97–60.34)
Increasing financial support for RD research	154	53.10 (47.24–58.97)
Ensuring more efficient communication between healthcare practitioners and lawmakers for legal regulations	148	51.03 (45.17–56.55)
Increasing collaboration between healthcare institutions and researchers	141	48.62 (42.76–54.14)
Improving insurance coverage for diagnosis and treatment	109	37.59 (32.07–43.10)
Providing/increasing incentives for pharmaceutical companies to develop orphan drugs	99	34.14 (28.62–39.66)
Increasing support for patient advocacy organizations and awareness campaigns	62	21.38 (16.90–26.21)
Popularizing telemedicine and telehealth services	47	16.21 (12.07–20.34)

Multiple options could be selected in this question (up to a maximum of 7). Response rate 79.89%.

## 4 Discussion

The findings of this study provide critical information on the current status of rare diseases (RD) research and management in Türkiye, highlighting key challenges and opportunities for improvement. In this context, the study was conducted with healthcare professionals and researchers who are integral stakeholders of the RD ecosystem, encompassing diagnosis, treatment, drug development and policy makers in this field. The majority of participants were mid-career professionals specializing in pediatrics and biology-genetics, aligning with the fact that RD frequently occurs in childhood and genetic factors play a significant role in these diseases (14).

In our survey, one of the most critical challenges encountered in the diagnosis, follow-up and management of RD is the necessity to refer patients to specialized centers for clinical evaluation and diagnostic tests. Furthermore, the primary challenges associated with patient follow-up can be attributed to two key factors: the necessity to refer patients to specialized centers and instances of patients discontinuing their follow-up appointments. Indeed, it is impractical to assess these factors independently. The necessity of referral to disparate medical centers is a globally pervasive issue encountered by patients afflicted with RD and the healthcare professionals who treat them (15, 16). A recent analysis revealed that a RD patient typically consults with six different medical professionals before receiving a definitive diagnosis and is subsequently referred to multiple hospitals over the course of several years (17). It has been documented that the average diagnosis time for children with RD in Australia is 18 years, with cases in Poland taking up to 30 years (18, 19). This difficult and long diagnostic journey for patients causes serious psychological pressure on both patients and their families. As a consequence of this exhausting process, overwhelmed patients may discontinue follow-up/care (17, 20). In order to address the challenges associated with the management of RD, the Ministry of Health in Türkiye has identified the establishment of specialized service units and centers of excellence as a key priority within the framework of the *“Rare Diseases Health Strategy Document and Action Plan* (*2023–2027*)*”* (21). One of the ultimate goals of the RareBoost project is to transform IBG, located in Izmir, one of Türkiye’s three largest cities, into a center of excellence in the field of RD by offering a multidisciplinary approach.

In contrast, challenges associated with patient follow-up stem primarily from instances where patients discontinue follow-up appointments. This discontinuation can be attributed to factors such as psychological fatigue, the logistical burden of repeated visits to specialized centers, or a lack of clear communication regarding the importance of long-term monitoring. Ensuring patients return for follow-ups requires a multifaceted strategy. First, establishing patient-centric care pathways within specialized centers can reduce logistical burdens by integrating multiple healthcare services in one location. Second, providing psychosocial support and patient education about the importance of follow-up appointments can address the psychological and informational gaps. Additionally, leveraging digital health technologies, such as telemedicine and electronic health record systems, can facilitate remote monitoring and reduce the necessity for frequent in-person visits, thereby improving adherence to follow-up care. Addressing both diagnostic and follow-up challenges in a coordinated manner is crucial to improving patient outcomes and quality of life for those with RD.

Another significant challenge arising from the survey related to the diagnostic process was a lack of access to diagnostic tests. Less than half of the respondents indicated that they had access to diagnostic tests supported by the Ministry of Health, while one-third reported that they had access to such tests with the support of drug companies or scientific projects. Those who indicated that they were unable to access diagnostic tests cited insufficient resources at their respective centers and the absence of test fee coverage under the general health insurance plan as the primary reasons. Türkiye’s state-sponsored health insurance program provides comprehensive coverage for all permanent residents under the age of 18, for health-related expenses. However, as shown in a recent study addressing the challenges faced by RD patients in Türkiye, health insurance appears to be insufficient to cover all medical expenses/tests (11). These challenges are present in Türkiye, as well as in Australia and the USA (18, 22).

From the perspective of health professionals and researchers, the most significant challenges in providing patients with access to care and treatment appear to be similar to those encountered in the diagnostic process: overwhelmed families, a lack of access to new treatments and inadequate insurance coverage of treatment or special education costs. Despite the existence of approximately 8,000 RD, the latest data indicates that there are only 882 FDA-approved (for 392 of these diseases) and 244 EMA-licensed orphan drugs (23, 24). It should also be noted that not all of these drugs are currently eligible for reimbursement in Türkiye (25). In Türkiye, many medicines imported from abroad are procured by the Ministry of Health. However, the situation changes when it comes to orphan drugs produced for RD. Due to the limited production of these medicines and some legal obstacles in reimbursement by the ministry, it is not always possible for those in need to access these medicines. A 2019 survey by the National Organization for Rare Diseases (NORD) revealed that 61% of US patients were denied or delayed by their insurance companies in accessing treatments that required prior authorization (prescriptions, medical devices, etc.) (26). It is unfortunate that access to new treatments and orphan drugs remains a major global challenge yet to be fully resolved.

When the challenges encountered in the field of RD management are analyzed from the perspective of various stakeholder groups, it reveals that researchers and healthcare professionals prioritize different issues. While researchers identify the lack of collaboration among themselves as a major problem, healthcare professionals highlight the length of time required to complete diagnostic tests as a major concern.

The generally low level of awareness of RD, when considered alongside the length and complexity of the diagnostic and treatment processes, represents a serious challenge to the effective management of these diseases (27). Although the majority of participants indicated that awareness of RD has increased in recent years, they expressed that this increase is still inadequate. A variety of initiatives have been implemented with the objective of disseminating training on a global scale, with a particular focus on raising awareness among health professionals and students in the field of RD. The German Academy for Further Medical Training on Rare Diseases (FAKSE) was established in Germany with the objective of building the awareness of professionals on the subject through video-based courses and case-based discussions with experts (28). Similarly, an RD training program for medical students has been established in Poland (29). As outlined in the *Rare Diseases Health Strategy Document and Action Plan* of the Ministry of Health in Türkiye, a training module for physicians on RD is currently being developed and is scheduled to launch in 2025, with targeted trainings to be completed by 2027 (21). It is also highlighted that collaboration with patient organizations and NGOs is an important strategy to spread awareness, provide education and support to healthcare professionals. Such collaborations not only increase awareness and participation in clinical research but also facilitate the development of innovative solutions and effective participation in policy development processes (30–32).

Despite the fact that the challenges encountered by the stakeholders in scientific and clinical research vary in their priorities, a number of common challenges have been identified as being of particular significance. These include the lack of public and private support for RD research, the absence of dialog, collaboration and data sharing, and the difficulty of conducting research with small patient cohorts. A recent study identifying research priorities for rare diseases in Ireland revealed that researchers were focusing on similar issues (33). The enhanced capabilities of disease mapping and population biobanks may facilitate resolution of the inherent limitation of small sample sizes associated with RD (34).

The participants in the study recommended that the most important policy improvements in the field of RD should be to increase national and international funding support for drugs and advanced research projects, establish follow-up systems for RD, and develop national action plans and guidelines on RD. These recommendations are consistent with the critical strategies of RD management frequently emphasized in the literature (15). Furthermore, it is recommended that prenatal screening coverage be expanded, and that more effective communication be established between healthcare providers and legislators. It is similarly recommended that collaboration between healthcare institutions and researchers be increased, that insurance coverage for diagnosis and treatment be improved, and that incentives be provided to pharmaceutical companies for the development of orphan drugs. A particular focus was placed on prenatal screening in order to ascertain the level of awareness among relevant healthcare professionals regarding genetic counseling for families. These recommendations are intended to facilitate more effective management of RD. Ultimately, the recommendations include increased support for patient advocacy organizations and awareness campaigns, as well as the expansion of telemedicine and telehealth services. These strategies and policy recommendations aim to fill existing gaps in this area and reflect a comprehensive approach to more effective management of RD and to improve the quality of life of patients affected by these diseases.

To the best of our knowledge, this is the first study to examine the obstacles and needs in the field of RD in Türkiye from the perspective of healthcare professionals and researchers. It should be noted, however, that the study is not without limitations. Firstly, the majority of participants in the study were healthcare professionals and researchers with a specialization in pediatrics and biology-genetics. This may restrict the scope for generalizing these findings. Secondly, although the proportion of respondents to the questions was generally high, there was considerable variation. It is of great importance that further research be conducted in order to determine the priorities in RD research in our country. Furthermore, it is of great importance that these efforts be supported by the state with a comprehensive and strategic approach. In addition, it should be noted that the challenges reported by participants regarding access to treatment, as shown in Figure 2, primarily focus on difficulties associated with accessing new treatment methods. The survey did not specifically address barriers to accessing current or existing treatment options, which represents a limitation of the study. The lack of specific geographical data of survey respondents limits the ability to analyze potential clustering of responses or gaps in collaboration and networking across Türkiye. It is clear that understanding the regional distribution of stakeholders would contribute to revealing the profiles of rare diseases in Türkiye.

This study sheds light on the principal challenges and prospects for enhancement in the diagnosis, treatment and management of RD in Türkiye. The findings highlight common issues, including the complexity of diagnostic and treatment processes, and the lack of access to diagnostic tests and advanced treatments. The study presents a series of recommendations, including the allocation of increased national and international funding for research and development, the establishment of more effective follow-up systems, and the enhancement of awareness among healthcare professionals. Such enhancements to the healthcare system are vital to improving the quality of life of patients affected by RD. We believe that these findings provide a guide to the steps to be followed for the dissemination of research in the field of RD.

## Data Availability

The raw data supporting the conclusions of this article will be made available by the authors, without undue reservation.

## Footnotes

^1^ https://shgmnadirdb.saglik.gov.tr

^2^ https://www.nadir.org.tr/en/

^3^ https://rareboost.ibg.edu.tr/
