# Supplementary material for: Management of rare and undiagnosed diseases: insights from researchers and healthcare professionals in Türkiye
Source: Front Public Health. 2025 Jan 15;12:1501942. doi: 10.3389/fpubh.2024.1501942 (PMC11795313; doi:10.3389/fpubh.2024.1501942)
Supplement: Supplementary file 1 [file Supplementary_file_1.docx]

*
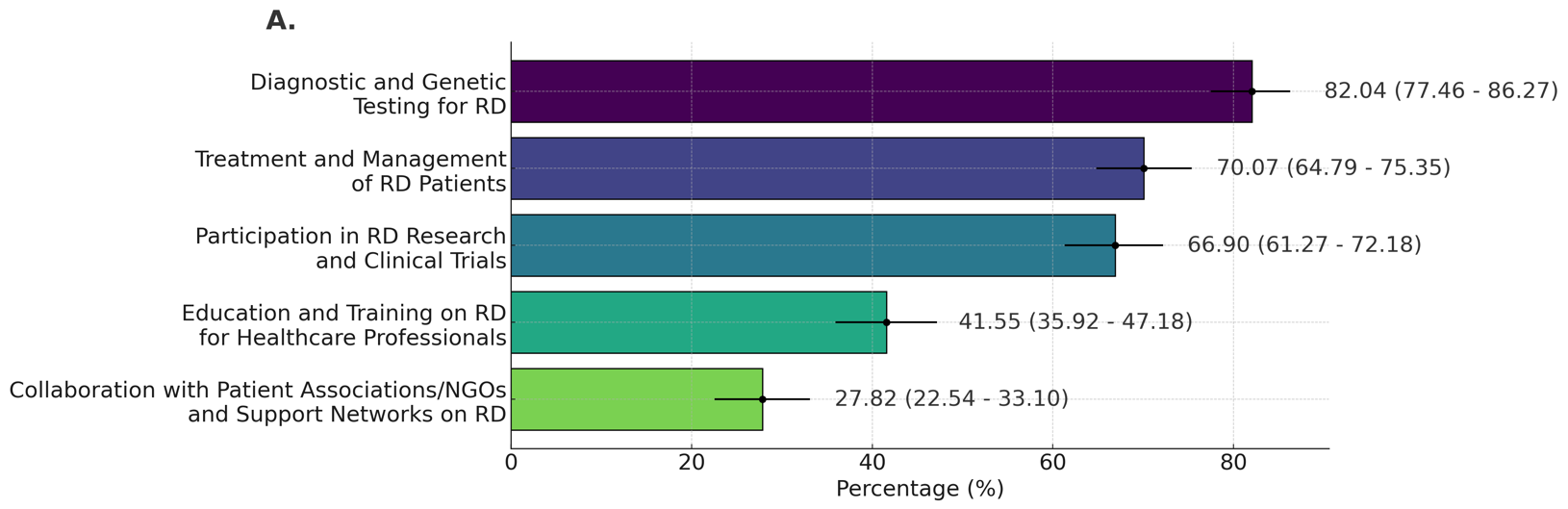
*

*
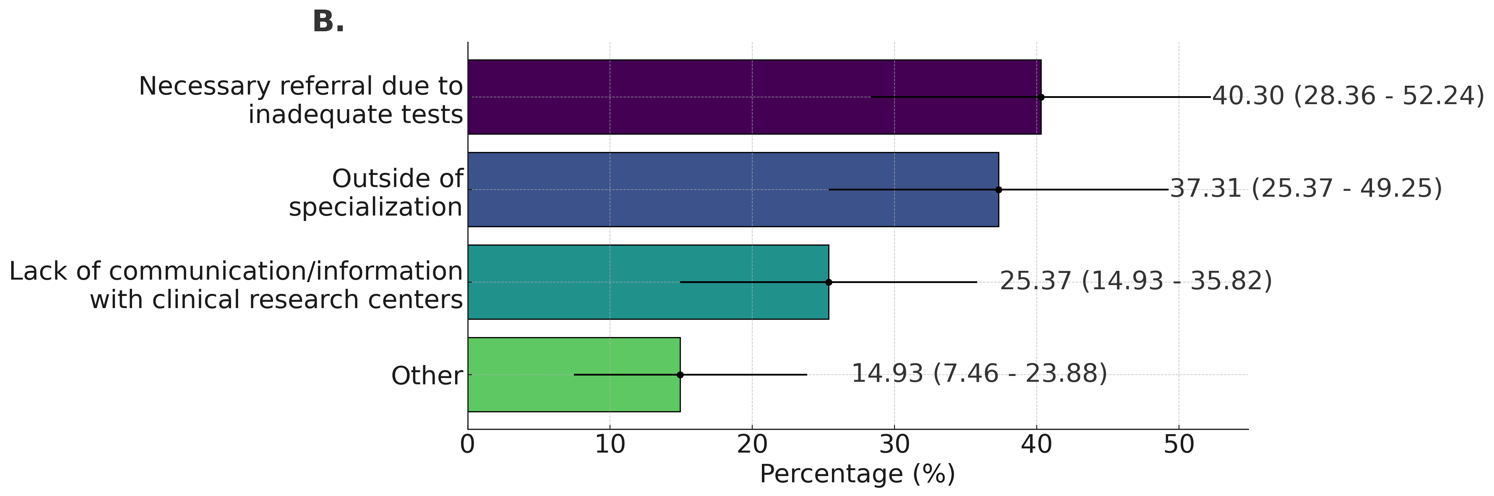
*

**Figure 1:** **A.** Activities in the field of RD (Q-10), **B.** Justifications for the lack of activity in the field of RD (Q-11).

*More than one answer option could be selected for the questions. Response rate for Q-10: 96.93%; for Q-11: 95.71%. Error bars indicating the 95% CIs.*

**TABLE 1: Distribution of Participants by Department**

| **Department** | **%** | **95% CI** |
| --- | --- | --- |
| Emergency Medicine | 0.28 | (0.01, 1.57) |
| Oral and Maxillofacial Surgery | 0.28 | (0.01, 1.57) |
| Oral and Maxillofacial Radiology | 0.28 | (0.01, 1.57) |
| Family Medicine | 3.03 | (1.68, 5.36) |
| Algology | 0.28 | (0.01, 1.57) |
| Anesthesiology and Reanimation | 0.28 | (0.01, 1.57) |
| Nutrition and Dietetics | 0.55 | (0.09, 1.96) |
| Neurosurgery | 0.28 | (0.01, 1.57) |
| Computer Science and Engineering | 0.55 | (0.09, 1.96) |
| Biology | 1.38 | (0.45, 3.18) |
| Biomedical Engineering | 2.75 | (1.32, 5.02) |
| Biostatistics | 0.55 | (0.09, 1.96) |
| Surgical Nursing | 0.55 | (0.09, 1.96) |
| Pediatric Endocrinology (Pediatrics) | 1.65 | (0.61, 3.61) |
| Pediatric Infectious Diseases (Pediatrics) | 0.28 | (0.01, 1.57) |
| Pediatric Gastroenterology (Pediatrics) | 0.28 | (0.01, 1.57) |
| Pediatric Genetic Diseases (Pediatrics) | 3.03 | (1.68, 5.36) |
| Pediatric Pulmonology (Pediatrics) | 1.10 | (0.36, 2.79) |
| Pediatric Hematology and Oncology (Pediatrics) | 10.74 | (8.00, 14.41) |
| Pediatric Immunology and Allergy Diseases | 0.83 | (0.21, 2.27) |
| Pediatric Cardiology (Pediatrics) | 0.55 | (0.09, 1.96) |
| Pediatric Metabolic Diseases (Pediatrics) | 2.48 | (1.28, 4.59) |
| Pediatric Neurology (Pediatrics) | 11.57 | (8.72, 15.26) |
| Pediatric Rheumatology (Pediatrics) | 2.20 | (1.05, 4.17) |
| Pediatrics | 1.65 | (0.61, 3.61) |
| Child and Adolescent Psychiatry | 0.55 | (0.09, 1.96) |
| Pediatric Intensive Care (Pediatrics) | 0.55 | (0.09, 1.96) |
| Internal Medicine Nursing | 0.28 | (0.01, 1.57) |
| Dermatology and Venereal Diseases | 0.28 | (0.01, 1.57) |
| Pharmaceutical Biochemistry | 0.28 | (0.01, 1.57) |
| Endocrinology and Metabolic Diseases | 2.20 | (1.05, 4.17) |
| Infectious Diseases and Clinical Microbiology | 0.28 | (0.01, 1.57) |
| Pharmacology | 0.55 | (0.09, 1.96) |
| Pharmaceutical Biotechnology | 0.55 | (0.09, 1.96) |
| Pharmaceutical Microbiology | 0.28 | (0.01, 1.57) |
| Pharmaceutical Toxicology | 0.28 | (0.01, 1.57) |
| Physical Medicine and Rehabilitation | 0.55 | (0.09, 1.96) |
| Physiology | 0.28 | (0.01, 1.57) |
| Physiotherapy and Rehabilitation | 0.55 | (0.09, 1.96) |
| Gastroenterology (Internal Medicine) | 0.28 | (0.01, 1.57) |
| Genomics | 1.93 | (0.78, 3.98) |
| Geriatrics (Internal Medicine) | 0.28 | (0.01, 1.57) |
| Pulmonology | 1.10 | (0.36, 2.79) |
| Public Health | 0.83 | (0.21, 2.27) |
| Hematology (Internal Medicine) | 0.28 | (0.01, 1.57) |
| Nursing | 0.55 | (0.09, 1.96) |
| Histology and Embryology | 0.55 | (0.09, 1.96) |
| Immunology and Allergy Diseases | 0.83 | (0.21, 2.27) |
| Gynecologic Oncology Surgery | 0.28 | (0.01, 1.57) |
| Obstetrics and Gynecology | 0.55 | (0.09, 1.96) |
| Cardiology | 0.28 | (0.01, 1.57) |
| Chemistry | 0.28 | (0.01, 1.57) |
| Clinical Pharmacy | 0.83 | (0.21, 2.27) |
| Clinical Neurophysiology | 0.55 | (0.09, 1.96) |
| Otorhinolaryngology | 0.28 | (0.01, 1.57) |
| Molecular Medicine | 1.10 | (0.36, 2.79) |
| Molecular Biology and Genetics | 9.64 | (6.89, 13.15) |
| Neonatology (Pediatrics) | 0.83 | (0.21, 2.27) |
| Neurology | 1.93 | (0.78, 3.98) |
| Orthodontics | 0.28 | (0.01, 1.57) |
| Orthopedics and Traumatology | 0.28 | (0.01, 1.57) |
| Periodontology | 0.55 | (0.09, 1.96) |
| Health Economics | 0.28 | (0.01, 1.57) |
| Health Management | 1.65 | (0.61, 3.61) |
| Neuroscience | 0.55 | (0.09, 1.96) |
| Basic Immunology | 0.83 | (0.21, 2.27) |
| Medical Biochemistry | 1.38 | (0.45, 3.18) |
| Medical Biology | 2.75 | (1.32, 5.02) |
| Medical Pharmacology | 0.55 | (0.09, 1.96) |
| Medical Genetics | 9.64 | (6.89, 13.15) |
| Medical Mycology (Medical Microbiology) | 0.28 | (0.01, 1.57) |
| Medical Oncology (Internal Medicine) | 0.28 | (0.01, 1.57) |
| Medical Pathology | 0.28 | (0.01, 1.57) |
| Medical Education | 0.28 | (0.01, 1.57) |
| Urology | 0.28 | (0.01, 1.57) |
| Veterinary Medicine | 0.28 | (0.01, 1.57) |
| Software Engineering | 0.28 | (0.01, 1.57) |
| Intensive Care | 0.28 | (0.01, 1.57) |

**TABLE 2: Challenges in Patient Care and Support for Patients and Their Families**

| ***Q22- Is support provided for the patient's needs during the diagnosis and follow-up process? (n=315)*** | **n** | **Distribution**  **% (95% CI)** |
| --- | --- | --- |
| *Yes* | 122 | 38.73 (33.35-44.11) |
| *No* | 68 | 21.59 (17.04-26.13) |
| *Don’t know* | 125 | 39.68 (34.28-45.09) |
| ***Q23- Support types for the patient's needs during the diagnosis and follow-up process (n=118)*** | **n** | **Distribution**  **% (95% CI)** |
| *Follow-up / Genetic Counseling* | 110 | 93.22 (88.68-97.76) |
| *Rehabilitation* | 72 | 61.02 (52.22-69.82) |
| *Communication with Representatives* | 34 | 28.81 (20.64-36.99) |
| *Specialized Clinics* | 81 | 68.64 (60.27-77.01) |

*Response rate* 86.78% *for Q22, 96.72% for Q23.*

**TABLE 3: Participants' Engagement in Scientific Research and Training in the Field of Rare and Undiagnosed Diseases**

| ***Q27- Involvement as a Researcher or Principal Investigator in Rare and Undiagnosed Diseases Research (n=307)*** | **n** | **Distribution**  **% (95% CI)** |
| --- | --- | --- |
| *Yes* | 178 | 57.98 (52.46-63.50) |
| *No* | 129 | 42.02 (36.50-47.54) |
| ***Q28- Involvement in Different Types of Research Related to Rare and Undiagnosed Diseases (n=178)*** | **n** | **Distribution**  **% (95% CI)** |
| *Diagnostic & Clinical Research* | 131 | 73.60 (67.12-80.07) |
| *Etiology Studies* | 69 | 38.76 (31.61-49.92) |
| *Treatment and Drug Development Research* | 55 | 30.90 (24.11-37.69) |
| *Disease Modeling* | 42 | 23.60 (17.36-29.83) |
| *Epidemiological Studies and Disease Prevalence* | 47 | 26.40 (19.93-32.88) |
| *Other* | 1 | 0.56 (0-1.66) |
| ***Q29-Training on the Diagnosis of RD During Education or Career (n=307)*** | **n** | **Distribution**  **% (95% CI)** |
| *Yes* | 203 | 66.12 (60.83-71.42) |
| *No* | 104 | 33.88 (28.58-39.17) |

*Participants were allowed to select more than one option for Q28. Response rate 84.57% for Q27 and Q28, 100% for Q23.*

**
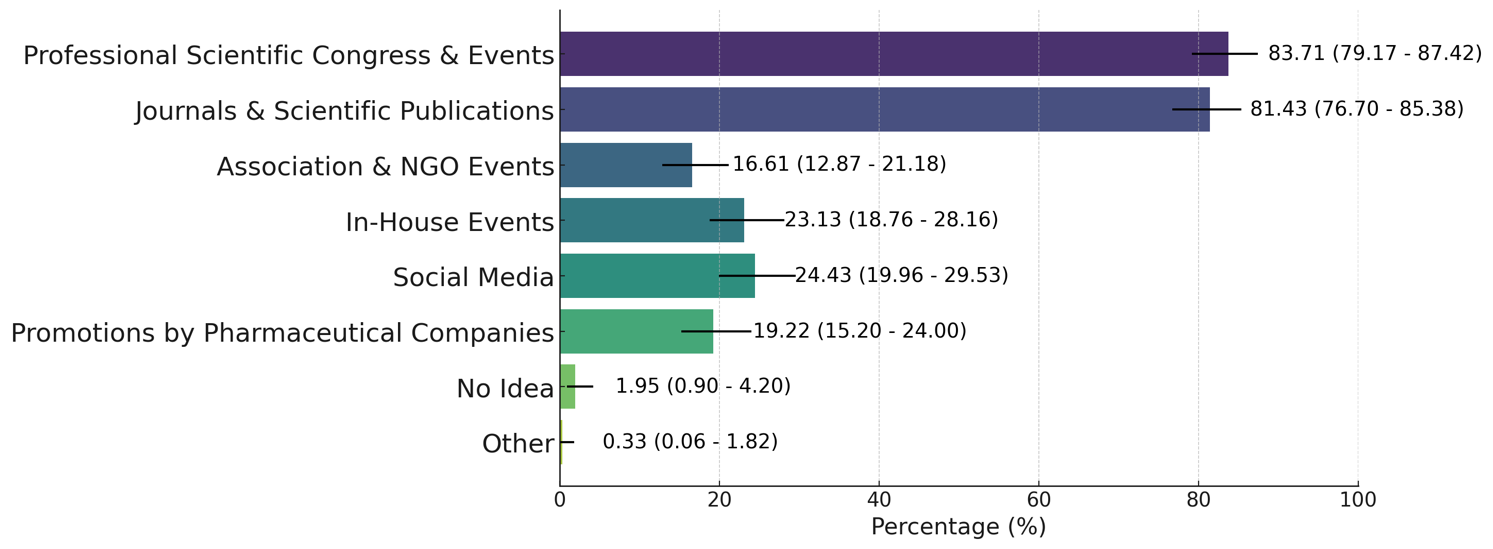
**

**Figure 2: Sources and methods of staying informed on the up to date in the field of RD (Q30)**

*Data are presented as percentages with 95%CI. Participants were allowed to select more than one option. Response rate: 84.57%.*

**
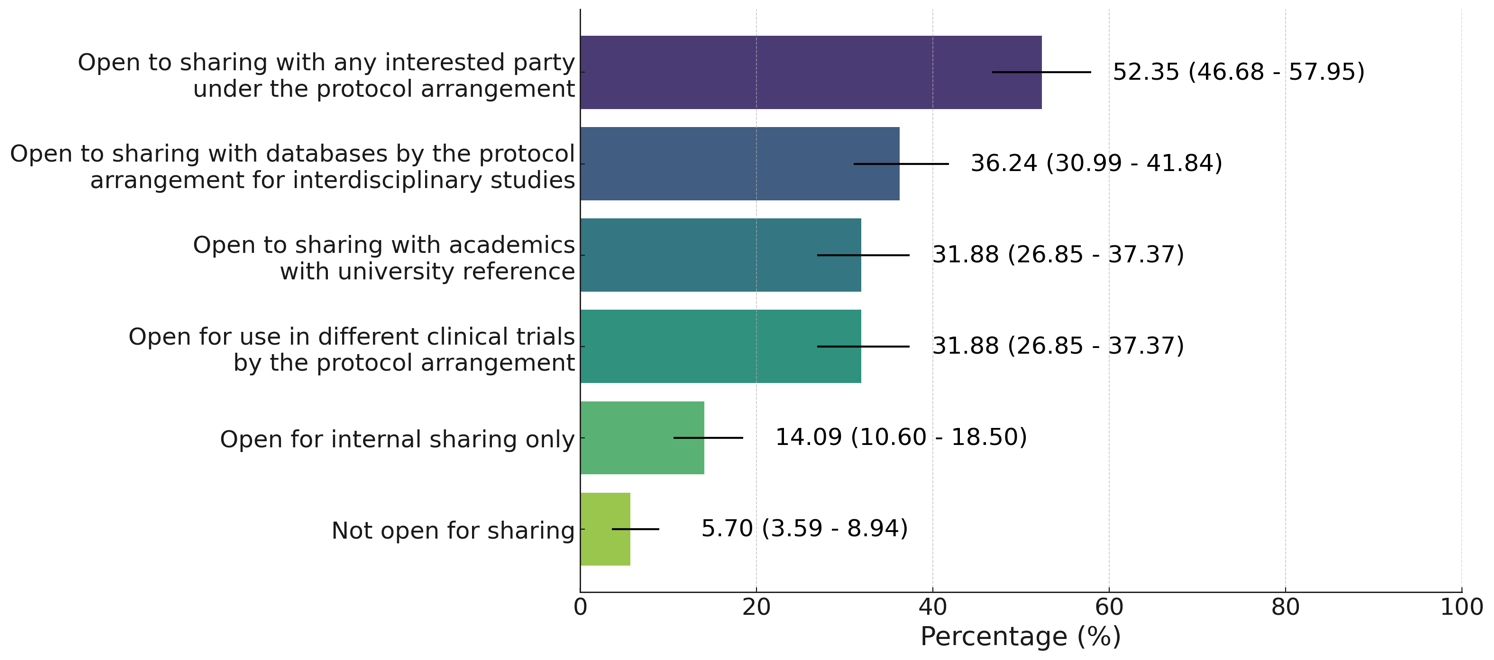
**

**Figure 3: The attitudes of the participants towards the sharing of data obtained from the sample used in their research (Q31)**

*Data are presented as percentages with 95%CI. Participants were allowed to select more than one option. Response rate: 82.09%.*

**TABLE 4: Ecosystem Stakeholders and Collaboration**

| ***Q33-Frequency of collaboration with other healthcare professionals in the diagnosis of RD (n=297)*** | **n** | **Distribution**  **% (95% CI)** |
| --- | --- | --- |
| *Daily* | 72 | 24.24 (19.72-29.43) |
| *Monthly* | 77 | 25.93 (21.27-31.19) |
| *Rarely* | 103 | 34.68 (29.49-40.26) |
| *Never* | 45 | 15.15 (11.52-19.67) |
| ***Q34-Involvement with or support for institutions, organizations, or NGOs related to rare and undiagnosed diseases, including collaboration, capacity-building activities, or providing assistance (n=297)*** | **n** | **Distribution**  **% (95% CI)** |
| *Yes* | 113 | 38.05 (32.71-43.69) |
| *No* | 184 | 61.95 (56.31-67.29) |
| ***Q35-Awareness of any government policy or initiative that supports research, treatment processes, patients, and their families related to rare and undiagnosed diseases (n=297)*** | **n** | **Distribution**  **% (95% CI)** |
| *Yes* | 89 | 29.97 (25.04-35.41) |
| *No* | 208 | 70.03 (64.59-74.96) |

*Response rate 81.82% for all.*
